# An incidental finding of low‐grade appendiceal mucinous neoplasm during cesarean section: A case report

**DOI:** 10.1002/jgh3.12232

**Published:** 2019-08-17

**Authors:** Eisuke Inubashiri, Yukio Watanabe, Noriyuki Akutagawa, Katumaru Kuroki, Masaki Sugawara, Keizou Deguchi, Nobuhiko Maeda, Fumitake Hata

**Affiliations:** ^1^ Sapporo Toho Hospital Sapporo Japan; ^2^ Sapporo Dohto Hospital Sapporo Japan

**Keywords:** colorectal cancer, clinical practice and treatment (including surgery), differentiation, gastroenterology, neoplasia

## Abstract

Low‐grade appendiceal mucinous neoplasms are rare and difficult to diagnose preoperatively because of a lack of characteristic symptoms. A 24‐year‐old female with no symptoms before pregnancy underwent an elective cesarean section at 38 weeks of gestation because of cephalo‐pelvic disproportion. Although no abnormalities were detected in the newborn, uterus, or uterine adnexa, a sausage‐like, swollen appendix was noted. The patient underwent appendectomy. Pathology showed the tumor was a low‐grade appendiceal mucinous neoplasm.

## Graphical abstract

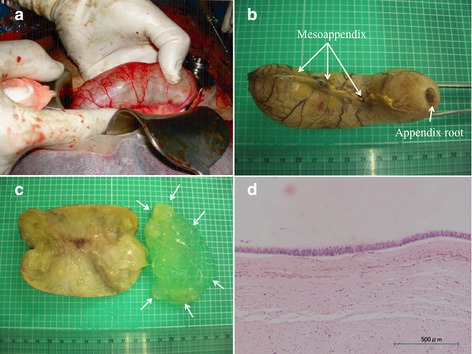

Here, we report the case of a 24‐year‐old pregnant women with incidentally discovered low‐grade appendiceal mucinous neoplasm (AMN) during cesarean section. To the best of our knowledge, there is a preoperatively diagnosed AMN complicating pregnancy. Previous reports suggested that AMN is detected during an operation performed for incidental reasons, such as acute abdomen, in all cases.

## Introduction

Appendiceal mucinous neoplasm (AMN) is a rare disease representing less than 0.3% of all appendectomy specimens.^1^ The appendiceal lumen dilates because of the accumulation of a mucinous substance.^1^, ^2^, ^3^, ^4^ These tumors cause the cystic expansion of the appendix through the accumulation of mucus in the lumen and can spread to the peritoneum, resulting in a specific syndrome called pseudomxyoma peritonei (PMP), which is often progressive and fatal.^3^ As both histologically benign and malignant AMNs can spread to the peritoneal cavity as PMP,^1^, ^3^, ^4^ a definitive diagnosis, including pathology and appropriate treatment, is important in AMNs.^1^, ^3^, ^4^, ^5^ No previous cases of low‐grade AMN (LAMN) during pregnancy have been reported. Here, we present the medical history of a patient with LAMN without PMP during pregnancy and briefly review the relevant literature.

## Clinical findings

**Figure 1:**
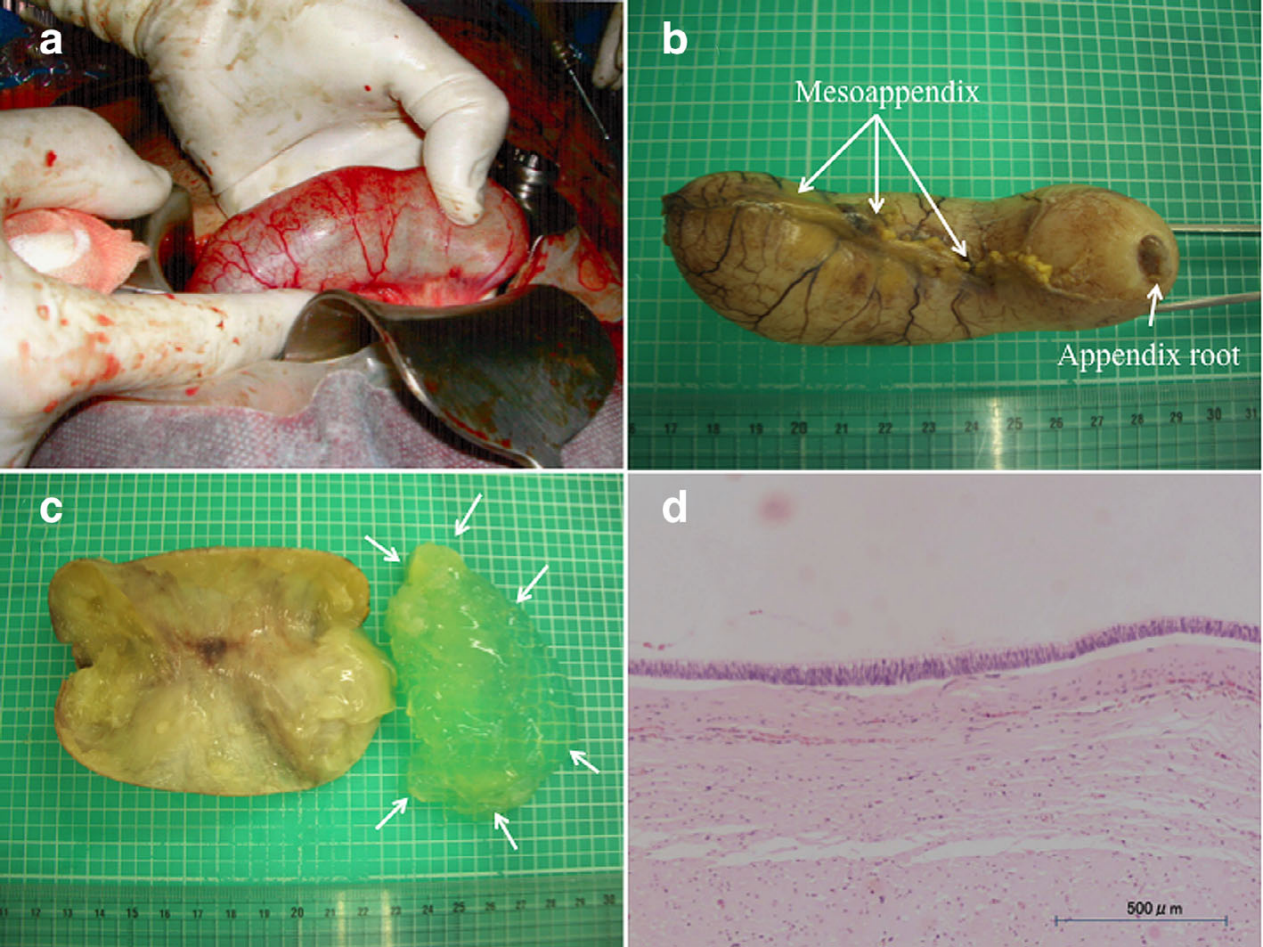
Photograph of the tumor during cesarean section (a). The resected low‐grade appendiceal mucinous neoplasm had a sausage‐like appearance (b). Panel (c) suggested that translucent mucosal substance was observed in the lumen of tumor (arrows). (d) The epithelium covering the lumen consisted of high columnar cells. The nuclei were oval‐shaped, and the cells were slightly stratified.

A 24‐year‐old female (gravida 0, para 0) had achieved spontaneous pregnancy and had been receiving pregnancy management as an outpatient at our hospital from the 6th week of gestation. No abnormality had been found in the sonography from the early stage of pregnancy, and the fetus had been developing normally. No particular problem occurred during the course of pregnancy. At 38 weeks of gestation, an elective cesarean section under combined spinal and epidural anesthesia was performed because of the indication of cephalopelvic disproportion. A female neonate weighing 3525 g and 49 cm in length was delivered uneventfully. The Apgar scores were 9 and 10 points at 1 and 5 min after birth, respectively. During the routine check at the time of the cesarean section, we confirmed that no abnormality was present in the uterus or uterine adnexa. However, the appendix was enlarged into a sausage‐like shape and possessed an elastic hardness (Fig. 1a,b). The root of the enlarged appendix was normal. We examined the abdominal cavity but found no other abnormalities (Fig. 1c). A general surgeon was immediately consulted and confirmed the high probability of an appendix tumor. We then performed ligation and disconnection of the mesoappendix and appendix root. After the subsequent removal of the appendix, we completed abdominal closure under combined spinal and epidural anesthesia. Routine laboratory examinations for cesarean section and routine prenatal sonography showed no abnormality. During pregnancy and prepregnancy, the patient had shown no preoperative symptoms (e.g. abdominal pain) suggesting appendicitis. Pathological examination showed an LAMN, which was equivalent to an adenoma. The tumor was restricted to the appendix, and the surgical margin was negative. The patient had an unremarkable postoperative course and was discharged home with her baby on the 6th postoperative day. We conducted a computed tomography (CT) scan for closer investigation at 1 month after the cesarean section and observed no metastasis or invasion. We plan to perform a careful follow‐up regularly with CT, ultrasound sonography (USG), and tumor markers for the next 5 years.

## Pathological findings

The appendix had a cyst‐like form and was 10 cm in length and 3 cm in diameter (Fig. 1b). The lumen was obstructed completely at the appendix root. After incision of the appendix, the tumor was found to be a unilocular cystic disease; the interior was filled with a white jelly‐like substance (Fig. 1c). The mucosal surface was edematous, but no protruded lesion was observed. The epithelium covering the lumen comprised high columnar cells possessing nuclei with an oval shape; only a slight degree of cellular stratification was present (Fig. 1d). No sign of mucosal fluid leakage into the interstitium or PMP was identified. The tumor had not ruptured and spilled mucin into the peritoneal cavity. Because no mucosal fluid leakage, no compelling cell stratification, and no severe cellular atypia were observed, we diagnosed the tumor as an LAMN.

## Discussion

LAMN can spread to the peritoneum as PMP.^1^, ^6^ Although PMP is not locally invasive, the mucin is locally destructive, and the resultant fibrosis and obstruction can lead to complications.^1^, ^3^, ^4^, ^7^ When restricted to the appendix, AMNs are indolent lesions with a 96% disease‐free survival at 5 years; however, when AMNs spread beyond the appendix, the disease‐free survival decreases to 66–67%^1^ Hence, the early detection of AMNs and appropriate treatment is very important for prognosis.^1^, ^3^, ^5^, ^8^, ^9^ Sugarbaker and Chang^4^ reported that patients with a complete cytoreduction and adenomucinosis by pathology had a 5‐year survival of 86%, and those with incomplete cytoreduction had a 5‐year survival of 20%. Tumors confined to the appendiceal mucosa were cured by appendectomy, whereas any proliferation of neoplastic epithelium beyond the mucosa places the patient at risk of peritoneal dissemination or adenocarcinoma and PMP. More aggressive treatment may be needed, which includes optimal debunking surgery and intraoperative intraperitoneal chemotherapy and additional cycles of postoperative chemotherapy.^1^, ^4^, ^7^ In our case, the tumor was confined to the appendix, and neoplasms were limited to mucosa. Based on low‐grade cytology, there was no spillage of mucosa. These histological findings indicated LAMN.^1^ PMP was also not noted in our case.

To our knowledge, there are eight previous reports of AMN during pregnancy.^5^, ^8^, ^9^ Four patients underwent laparotomy because of an acute abdomen during pregnancy; mucinous adenocarcinoma was diagnosed in each case.^8^ Meanwhile, two other patients showed no symptoms, but a right adnexal mass was noted as an incidental finding on obstetric sonography until the second trimester, when surgery was performed.^8^, ^9^ Of these two patients, one had appendiceal mucinous adenocarcinoma, while the other had appendiceal mucinous adenoma with PMP.^8^ Furthermore, two patients had tumors incidentally found on cesarean section.^8^ Mucinous adenocarcinoma was diagnosed in each case.^5^, ^8^ Our case was diagnosed on cesarean section, and the tumor was histologically found to be an LAMN without PMP. Clinical manifestations of LAMN without PMP may include pain in the right lower quadrant of the abdomen, palpable mass, nausea, vomiting, gastrointestinal bleeding, and signs of intestinal intussusception.^1^, ^7^ The differential diagnosis of abdominal pain in the right lower quadrant includes a wide variety of alternatives, such as appendicitis, appendiceal abscess, hydrosalpinx, ovarian tumor, endometrial cysts, and enterocolitis in female patients.^8^ In particular, obstetrical symptoms (e.g. uterine contraction) can obscure clinical manifestations of an appendiceal lesion in pregnant women.^8^, ^10^ However, the previous report of AMNs in pregnant women indicate that clinical manifestations such as acute abdomen can be a carcinoma.^5^, ^8^, ^9^ Collectively, these facts suggest the extreme difficulty in preoperatively diagnosing AMNs during pregnancy.

USG, CT, and magnetic resonance imaging (MRI) are reported to be useful in the preoperative imaging diagnosis of AMN.^1^, ^7^, ^8^, ^11^ However, in pregnant women, CT and MRI present the problem of a potentially harmful effect on the fetus, limiting their use.^2^ USG is thought to present no problems to pregnant women, but the pregnancy can make it difficult for both transabdominal and transvaginal USG to detect an appendiceal lesion, depending on the gestational age.^5^, ^8^, ^9^ As pointed out by Erika *et al*.,^8^ the diseases of the ileocecal region (e.g. ovarian cysts) are far more frequent in females than AMNs, and therefore, in many cases, AMNs are diagnosed not preoperatively but postoperatively.^8^ Another possibility for detecting AMNs are tumor markers. In particular, the tumor markers carcinoembryonic antigen (CEA) and carbohydrate antigen 19‐9 (CA 19‐9) are known to be elevated in AMNs^11^ and to show little change even at pregnancy.^12^ Hence, these markers have the potential to be useful in making a preoperative diagnosis and in evaluating progression and recurrence. In our case, both CEA and CA19‐9 indicated no abnormality postoperatively at 2 weeks.

Recently, a study showed that LAMN is divided into two subtypes: one type is confined to the appendiceal lumen, while the other type occurs as mucin and/or neoplastic epithelium in the appendiceal submucosa, wall, and/or periappendiceal tissue, with or/without perforation.^6^ The author concluded that the latter has a greater risk of progression and should be treated with cytoreductive surgery and chemotherapy.^6^ According to a population‐based study in the Netherlands for benign AMN, the association with PMP was 2%.^3^ Together, these studies suggest that careful follow‐up might be needed even in cases of benign LAMN. For AMNs, aggressive treatment, including optimal debunking surgery and/or chemotherapy, is needed.^1^, ^3^, ^4^, ^6^, ^7^

To our knowledge, there is no report of a preoperatively diagnosed AMN complicating pregnancy. In all cases, AMN was detected during an operation performed for incidental reasons, such as acute abdomen or an adnexal mass.^5^, ^8^, ^9^ Although appendicitis, ovarian tumor, hydrosalpinx, etc. are considered to be more frequent in females, the possibility of AMN should potentially be taken into account in the future. Based on the present case and the previous case reports, we suggest that not only should routine check‐ups of the uterus or uterine adnexa be performed but that an examination of the pelvic viscera (including the appendix) during a cesarean section can offer an opportunity for the definite diagnosis of occult cancer or benign tumors such as AMN.

## References

[1] Panarelli NC , Yantiss RK . Mucinous neoplasms of the appendix and peritoneum. Arch. Pathol. Lab. Med. 2011; 135: 1261–8. 21970481 10.5858/arpa.2011-0034-RA

[2] Chen MM , Coakley FV , Kaimal A , Laros RK Jr . Guidelines for computed tomography and magnetic resonance imaging use during pregnancy and lactation. Obstet. Gynecol. 2008; 112(2, Part 1): 333–40. 18669732 10.1097/AOG.0b013e318180a505

[3] Smeenk RM , Van Velthuysen MLF , Verwaal VJ , Zoetmulder FAN . Appendiceal neoplasms and pseudomyxoma peritonei: a population based study. Eur. J. Surg. Oncol. 2008; 34: 196–201. 17524597 10.1016/j.ejso.2007.04.002

[4] Sugarbaker PH , Chang D . Results of treatment of 385 patients with peritoneal surface spread of appendiceal malignancy. Ann. Surg. Oncol. 1999; 6: 727–31. 10622499 10.1007/s10434-999-0727-7

[5] Abdu B , Hobgood D , Stallings S , Depasquale S . Incidental finding of pseudomyxoma peritonei at primary cesarean section. Am. J. Perinatol. 2009; 26: 633–5. 19399708 10.1055/s-0029-1220791

[6] McDonald JR , O'Dwyer ST , Rout S *et al* Classification of and cytoreductive surgery for low‐grade appendiceal mucinous neoplasms. Br. J. Surg. 2012; 99: 987–92. 22517234 10.1002/bjs.8739

[7] Andreopoulou E , Yee H , Warycha M *et al* Mucinous cancer of the appendix: challenges in diagnosis and treatment. J. Chemother. 2007; 19: 451–4. 17855191 10.1179/joc.2007.19.4.451

[8] Erika H , Dal Y , Paul S . Management of appendiceal pseudomyxoma peritonei diagnosed during pregnancy. World J. Surg. Oncol. 2009; 7: 48. 19454019 10.1186/1477-7819-7-48 PMC2688488

[9] Kalu E , Croucher C . Appendiceal mucocele: a rare differential diagnosis of a cystic right adnexal mass. Arch. Gynecol. Obstet. 2005; 271: 86–8. 15316825 10.1007/s00404-004-0663-5

[10] Andersen B , Nielsen T . Appendicitis in pregnancy, Diagnosis, management and complications. Acta Obstet. Gynecol. Scand. 1999; 78: 758–62. 10535336

[11] Demetrashvili Z , Chkhaidze M , Khutsishvili K *et al* Mucocele of the appendix: case report and review of literature. Int. Surg. 2012; 97: 266–9. 23113858 10.9738/CC139.1 PMC3723229

[12] Ercan Ş , Kaymaz Ö , Yücel N , Orçun A . Serum concentrations of CA 125, CA 15‐3, CA 19‐9 and CEA in normal pregnancy: a longitudinal study. Arch. Gynecol. Obstet. 2012; 285: 579–84. 21792548 10.1007/s00404-011-2025-4

